# Identification of Mepenzolate Derivatives With Long-Acting Bronchodilatory Activity

**DOI:** 10.3389/fphar.2018.00344

**Published:** 2018-04-10

**Authors:** Ken-Ichiro Tanaka, Naoki Yamakawa, Yasunobu Yamashita, Teita Asano, Yuki Kanda, Ayaka Takafuji, Masahiro Kawahara, Mitsuko Takenaga, Yoshifumi Fukunishi, Tohru Mizushima

**Affiliations:** ^1^Research Institute of Pharmaceutical Sciences, Faculty of Pharmacy, Musashino University, Nishi-Tokyo, Japan; ^2^School of Pharmacy, Shujitsu University, Okayama, Japan; ^3^Molecular Profiling Research Center for Drug Discovery, National Institute of Advanced Industrial Science and Technology, Tokyo, Japan; ^4^Institute of Medical Science, School of Medicine, St. Marianna University, Kawasaki, Japan; ^5^LTT Bio-Pharma Co., Ltd., Tokyo, Japan

**Keywords:** chronic obstructive pulmonary disease, anti-inflammatory, long-acting bronchodilatory activity, mepenzolate

## Abstract

The standard treatment for chronic obstructive pulmonary disease is a combination of anti-inflammatory drugs and bronchodilators. We recently found that mepenzolate bromide (**MP**), an antagonist for human muscarinic M3 receptor (hM_3_R), has both anti-inflammatory and short-acting bronchodilatory activities. To obtain **MP** derivatives with longer-lasting bronchodilatory activity, we synthesized hybrid compounds based on **MP** and two other muscarinic antagonists with long-acting bronchodilatory activity glycopyrronium bromide (**GC**) and aclidinium bromide (**AD**). Of these three synthesized hybrid compounds (**MP-GC, GC-MP, MP-AD**) and **MP, MP-AD** showed the highest affinity for hM_3_R and had the longest lasting bronchodilatory activity, which was equivalent to that of **GC** and **AD**. Both **MP-GC** and **MP-AD** exhibited an anti-inflammatory effect equivalent to that of MP, whereas, in line with **GC** and **AD, GC-MP** did not show this effect. We also confirmed that administration of **MP-AD** suppressed elastase-induced pulmonary emphysema in a mouse model. These findings provide important information about the structure-activity relationship of **MP** for both bronchodilatory and anti-inflammatory activities.

## Introduction

Chronic obstructive pulmonary disease (COPD) is a serious health problem and the world’s fourth leading cause of death (Mannino and Kiriz, 2006). This disease state is defined by a progressive and not fully reversible airflow limitation and an abnormal inflammatory response (Rabe et al., 2007; Vestbo et al., 2013; Vogelmeier et al., 2017). For the clinical treatment of COPD, it is therefore important not only to improve airflow limitations by inducing bronchodilation, but also to hinder disease progression by suppressing inflammatory responses.

The standard treatment for COPD is to use bronchodilators such as β2-agonists and muscarinic receptor antagonists, with this approach being effective in overcoming airflow limitations (Barnes and Stockley, 2005; Vestbo et al., 2013; Vogelmeier et al., 2017). Short-acting β2-agonists (SABAs) and muscarinic antagonists (SAMAs) were previously used for COPD treatment; however, long-acting β2-agonists (LABAs) and muscarinic antagonists [LAMAs, of which tiotropium bromide (**TP**), glycopyrronium bromide (**GC**), and aclidinium bromide (**AD**) are common examples] have shown superior efficacy over the short-acting bronchodilators for the treatment of COPD (Vestbo et al., 2013; Vogelmeier et al., 2017). Thus, LABAs and LAMAs (or combinations of the two) are now used as first-line drugs for COPD treatment.

On the other hand, the inflammatory responses associated with COPD are generally treated with steroids; however, their impact on reducing disease progression and mortality (Barnes and Stockley, 2005; Calverley et al., 2007; Tashkin et al., 2008) is attenuated because COPD-associated inflammation often shows resistance to steroid treatment (Alsaeedi et al., 2002; Calverley et al., 2007). Thus, it is important for new types of anti-inflammatory compounds to be developed for the treatment of COPD.

Based on the aforementioned, we previously screened a library of approved medicines to identify compounds capable of preventing elastase-induced pulmonary emphysema in mice (an animal model of COPD), and selected mepenzolate bromide (**MP**) (Tanaka et al., 2013), which is an orally administered muscarinic receptor antagonist used to treat gastrointestinal disorders (Long and Keasling, 1954; Buckley et al., 1957; Chen, 1959). We showed that **MP** not only exerts an anti-inflammatory effect via a muscarinic receptor-independent mechanism, but also possesses a short-acting bronchodilatory effect via a muscarinic receptor-dependent mechanism [via its binding to human muscarinic M3 receptor (hM_3_R)] (Tanaka et al., 2013, 2014; Kurotsu et al., 2014). The anti-inflammatory activity of **MP**, which was more potent than that of steroids (Tanaka et al., 2012, 2013), is mediated via a decreased level of reactive oxygen species (ROS) in the lung due to the inhibition of NADPH oxidase activity and the induction of superoxide dismutase (SOD) and glutathione-*S*-transferase (GST) expression (Tanaka et al., 2013).

In the present study, we examined **MP** derivatives that have both anti-inflammatory and longer-acting bronchodilatory activities, as these could be beneficial for the treatment of COPD. To this end, we synthesized hybrid compounds from **MP** and long-acting muscarinic antagonists (**GC** and **AD**) and identified one compound that showed both a longer duration of bronchodilatory activity (equivalent to **GC** and **AD**) together with anti-inflammatory properties consistent with those of **MP**.

## Materials and Methods

### Chemistry

Muscarinic M3 receptor antagonists were purchased from commercial sources; mepenzolate bromide **(MP)** was from Sigma-Aldrich (St. Louis, MO, United States); tiotropium bromide (**TP**) and glycopyrronium bromide (**GC**) were from Tokyo Chemical Industry, Co., Ltd. (Tokyo, Japan); aclidinium bromide (**AD**) was from Toronto Research Chemicals (Toronto, ON, Canada). All other solvents and reagents for organic syntheses were purchased from Tokyo Chemical Industry, Co., Ltd. (Tokyo, Japan) or Wako Pure Chemical Industries (Tokyo, Japan) and used without further purification. The precursor of hybrid compound (**pre-Hyb**) was synthesized by the condensation of carboxylic acid (**BA** or **CA**) and amino-alcohol (**PR, PP**, or **QC**). The each final quaternary salt of hybrid compounds (**MP-GC, GC-MP, MP-AD**) was prepared as described previously (Yamashita et al., 2014), with some modifications (see below). Column chromatography was performed using Silica gel 60N (Kanto Chemical, Co., Tokyo, Japan).

Melting point (mp) data were recorded on Yanaco MP-J3 micro-melting point apparatus. ^1^H-NMR spectra and ^13^C-NMR spectra were measured at 400 and 100 MHz, respectively, on a VARIAN 400-MR spectrometer. High resolution mass spectra were detected with an ESI-TOF mass spectrometer (Bruker MicroTOF, Bruker, Bremen, Germany) in the positive mode. HPLC data were recorded on a Waters 2695 Alliance separation module and a Waters 2996 photodiode array detector (Waters, Milford, MA, United States) with a reverse-phase column (TSKgel Super-ODS, 150 mm × 4.6 mm, 2 μm, Tosoh, Co., Tokyo, Japan). CH_3_CN/20 mM KH_2_PO_4_ buffer (3:7) was used as the mobile phase at a flow rate of 0.3 ml/min, with detection performed at an optical density of 210 nm.

### General Procedure for the Preparation of pre-Hyb

To a solution of 2-alkyl-2-hydroxy-2-phenylacetic acid (**BA** or **CA**) (1.0 eq.) in DMF, carbonyldiimidazole (1.6 eq.) was added and the mixture was stirred for 15 min at room temperature. A solution of *N*-alkyl amino alcohol (**PR, PP**, or **QC**) (1.2 eq.) in DMF was added dropwise into the mixture at 80°C and the mixture was stirred for 18 h at that temperature. The reaction was quenched with H_2_O at room temperature and organic materials were extracted with EtOAc. The combined extract was washed with brine, dried over Na_2_SO_4_ and concentrated *in vacuo*. The residue was then applied to a short silica gel column, eluted with EtOAc and concentrated *in vacuo* to give title compound (**pre-Hyb**), which was used for the next step without further purification.

#### (±)-3-(2-Hydroxy-2,2-diphenylacetoxy)-1,1-dimethylpyrrolidinium Bromide (MP-GC)

2.0 g (8.7 mmol) of benzilic acid **(BA)** and 1.3 mL (11 mmol) of 1-methylpyrrolidin-3-ol (**PR**) were used to give **pre-Hyb**. To a solution of **pre-Hyb** (2.2 g, 7.0 mmol) in CH_3_CN (12 mL), CH_3_Br (2.0 M in THF, 18 mL) was added and stirred for 5 h at room temperature. The precipitate was filtered off and the filtrate re-crystallized with CH_3_CN and diethyl ether to give the title compound as a white solid (2.0 g, 69% 2 steps). mp: 211–212°C; ^1^H-NMR (DMSO-*d*6): δ = 7.29–7.38 (m, 10H), 6.81 (s, 1H), 5.51 (brs, 1H), 3.84–3.89 (m, 1H), 3.52–3.70 (m, 3H), 3.6 (s, 3H), 2.93 (s, 3H), 2.62–2.74 (m, 1H), 2.08–2.14 (m, 1H); ^13^C-NMR (DMSO-*d*6): *d* = 172.3, 142.9, 127.9, 127.7, 127.0, 80.6, 73.3, 69.3, 64.0, 52.7, 51.9, 40.2, 38.9, 29.9; HRMS: calcd. for C_20_H_24_NO_3_ (M^+^): 326.1751; found: m/z = 326.1755; HPLC: *t*_R_ (min) = 3.12 (98%). This is a known compound, but its spectrum data are not covered by patents or non-patent literature documents.

#### (±)-3-(2-Cyclopentyl-2-hydroxy-2-phenylacetoxy)-1,1-dimethylpiperidinium Bromide (GC-MP)

1.5 g (6.8 mmol) of 2-cyclopentyl-2-hydroxy-2-phenylacetic acid (**CA**) and 1.0 mL (8.1 mmol) of 1-methylpiperidin-3-ol (**PP**) were used to give **pre-Hyb**. To a solution of **pre-Hyb** (0.41 g, 1.3 mmol) in CH_3_CN (5 mL), CH_3_Br (2.0 M in THF, 6.4 mL) was added and stirred for 5 h at room temperature. The precipitate was filtered off and the filtrate re-crystallized with CH_3_CN and diethyl ether to give the title compound as a white solid (0.38 g, 22% 2 steps). mp: 164–165°C; ^1^H-NMR (DMSO-*d*6): δ = 7.56–7.59 (d, *J* = 7.5, 2H), 7.25–7.37 (m, 3H), 5.14 (brs, 1H), 3.37–3.62 (m, 4H), 2.89–3.06 (m, 7H), 1.23–1.79 (m, 12H); ^13^C-NMR (DMSO-*d*6): δ = 173.0, 142.2, 142.7, 128.0, 127.3, 125.7, 79.3, 66.1, 61.5, 60.8, 46.4, 40.1, 38.9, 26.6, 26.0, 25.6, 25.4, 16.4; HRMS calcd. for C_20_H_30_NO_3_ (M^+^): 332.2220; found: m/z = 332.2224; HPLC: *t*_R_ (min) = 4.94 (98%). This is a known compound, but its spectrum data are not covered by patents or non-patent literature documents.

#### (±)-3-(2-Hydroxy-2,2-diphenylacetoxy)-1-(3-phenoxypropyl)-1-azoniabicyclo[2.2.2]octane Bromide (MP-AD)

1.6 g (0.60 mmol) of benzilic acid (**BA**) and 1.0 g (7.9 mmol) of quinuclidin-3-ol (**QC**) were used to give **pre-Hyb**. To a solution of **pre-Hyb** (0.10 g, 0.30 mmol) in 1,4-dioxane (5 mL) (3-bromopropoxy)benzene (0.11 g, 11 mmol) was added and stirred for 6 days at room temperature. The precipitate was filtered off and the filtrate re-crystallized with CH_3_CN and diethyl ether to give the title compound as a white solid (50 mg, 30% 2 steps). mp: 177–178°C; ^1^H-NMR (DMSO-*d*6): δ = 7.23–7.42 (m, 12H), 6.86–6.89 (m, 3H), 6.78 (s, 1H), 5.22 (brs, 1H), 3.94–4.04 (m, 2H), 3.85–3.88 (m, 1H), 3.25–3.44 (m, 5H), 3.01–3.07 (m, 1H), 1.45–2.25 (m, 7H); ^13^C-NMR (DMSO-*d*6): δ = 173.0, 158.6, 143.6, 143.5, 130.1, 128.5, 128.4, 128.2, 127.6, 127.5, 121.5, 115.0, 81.3, 69.1, 65.1, 61.1, 60.0, 54.3, 53.2, 24.0, 22.2, 20.9, 18.1; HRMS calcd. for C_30_H_34_NO_4_ (M^+^): 472.2482; found: m/z = 472.2488; HPLC: *t*_R_ (min) = 9.26 (99%). This is a known compound whose ^1^H-NMR spectrum was similar to that previously reported as (*R*)-**MP-GC** (Yamashita et al., 2014).

### Chemicals

Porcine pancreatic elastase (PPE) and methacholine chloride (methacholine) were obtained from Sigma-Aldrich (St. Louis, MO, United States). Novo-Heparin for injection was from Mochida Pharmaceutical, Co. (Tokyo, Japan). Chloral hydrate was from Nacalai Tesque (Kyoto, Japan). Diff-Quik was from Sysmex, Co. (Kobe, Japan). Formalin neutral buffer solution was from WAKO Pure Chemicals (Tokyo, Japan). Mayer’s hematoxylin, 1% eosin alcohol solution and malinol were from MUTO Pure Chemicals (Tokyo, Japan).

### Animals

ICR mice (6–7 weeks old, male) were purchased from Charles River (Yokohama, Japan). The experiments and procedures described here were carried out in accordance with the Guide for the Care and Use of Laboratory Animals as adopted and promulgated by the National Institutes of Health, and were approved by the Animal Care Committee of Musashino University (Approval NOs. 16004 and 17006).

### Treatment of Mice With PPE and Each Drug

Mice maintained under anesthesia with isoflurane were administered one intratracheal bolus of PPE (15 or 20 U/kg), **MP** (3.8, 37.5 μg/kg), **TP** (8.4 μg/kg), **GC** (7.1, 35.5 μg/kg), **AD** (10.1, 50.4 μg/kg), **MP-GC** (3.6, 36.2 μg/kg), **GC-MP** (3.7, 36.8 μg/kg), and **MP-AD** (4.9, 49.3 μg/kg) in 0.9% NaCl via micropipette (P200). For control mice, 0.9% NaCl alone was administered by the same procedure.

**Figure 3**; MP, **GC, AD, MP-AD, MP-GC**, or **GC-MP** were administered intratracheally. After 1, 24, 48, or 72 h, mice were exposed to nebulized methacholine 5 times and airway resistance was determined after each methacholine challenge.

**Figure 4** and **Table 2**; **MP, TP, GC, AD, MP-AD, MP-GC**, and **GC-MP** were administered intratracheally once only. After 1 h, mice were treated with or without (control) PPE (20 U/kg) once only. Six (**Figure 4**) or twenty-four (**Table 2**) hours after the PPE administration, BALF was prepared and the total cell number and the number of neutrophils were determined.

**Figure 5**; MP-AD was administered intratracheally once daily for 16 days (from day 0 to day 15). 1 h after the first administration, mice were treated with or without (control) PPE (15 U/kg) once only on day 0. 21 days after the PPE administration, lung mechanics was monitored, and sections of pulmonary tissue were prepared.

### Preparation of Bronchoalveolar Lavage Fluid (BALF) and Cell Count Method

BALF was collected from mice by cannulating the trachea and lavaging the lung with 1 ml of sterile PBS containing 50 U/ml heparin (2 times). About 1.8 ml of BALF was routinely recovered from each animal. The total cell number in the BALF was counted using a hemocytometer and a manual counter. Cells were stained with Diff-Quik reagents after centrifugation with Cytospin^®^ 4 (Thermo Electron Corporation, Waltham, MA, United States), and differential counts of more than 50 inflammatory cells were done in each of four or more separate sections of smear using fluorescence microscopy (Olympus DP71). The ratio of number of neutrophils to total cell number was determined by counting the number of neutrophils. This cell counting was conducted by an investigator blinded to the study protocol.

### Measurement of Lung Mechanics and Airway Resistance

Lung mechanics and airway resistance were monitored with a computer-controlled small-animal ventilator (FlexiVent, SCIREQ, Montreal, QC, Canada), as described previously (Shalaby et al., 2010; Tanaka et al., 2012). Mice were anesthetized with chloral hydrate (500 mg/kg), a tracheotomy was performed, and an 8 mm section of metallic tube was inserted into the trachea. Mice were mechanically ventilated at a rate of 150 breaths/min, using a tidal volume of 8.7 ml/kg and a positive end-expiratory pressure of 2–3 cmH_2_O.

Total respiratory system compliance was measured by the snapshot. All data were analyzed using FlexiVent software (version 5.3; SCIREQ, Montreal, QC, Canada).

Mice were exposed to nebulized methacholine (5 mg/ml) five times for 20 s with a 40 s interval, and airway resistance was measured after each methacholine challenge by the snapshot technique. All data were analyzed using FlexiVent software.

### Histopathological Analysis

Twenty one days after PPE treatment, mice were euthanized with an excess of isoflurane. Lung tissue samples were fixed in 10% formalin neutral buffer solution for 24 h at a pressure of 25 cmH_2_O, and then embedded in paraffin before being cut into 4 μm-thick sections. Sections were stained first with Mayer’s hematoxylin and then with 1% eosin alcohol solution (H&E staining). Samples were mounted with malinol and scanned using a NanoZoomer-XR digital slide scanner (Hamamatsu Photonics, Shizuoka, Japan). All images were analyzed using NDP.view2 software (Hamamatsu Photonics, Shizuoka, Japan).

To determine the mean linear intercept (MLI) (an indicator of airspace enlargement), 20 lines (800 μm) were drawn randomly on the image of a section and intersection points with alveolar walls were counted to determine the MLI, as described previously (Weibel, 1963). This morphometric analysis was conducted by an investigator blinded to the study protocol.

### Filter-Binding Assay

The filter-binding assay was performed as described previously (Yamashita et al., 2014), with some modifications. Membrane fractions prepared from Chinese Hamster Ovary (CHO)-K1 cells expressing human muscarinic M2 receptor (hM_2_R) or hM_3_R (Membrane Target Systems, Perkin-Elmer Life and Analytical Sciences, Boston, MA, United States; protein concentration, 8 μg/well) were incubated with 2 nM [^3^H]NMS (*N*-methyl-[^3^H]-scopolamine methylchloride) (85.5 Ci/mmol) at room temperature for 2 h in 200 μL PBS in the presence of each compound. A range of concentrations (10^-11^ to 10^-6^ M) for each compound was tested in triplicate to generate competitive binding curves. Non-specific binding was determined in the presence of atropine (2.5 μM). The samples were passed through a GF/C filter (Filtermat A, PerkinElmer Life and Analytical Sciences, Boston, MA, United States) pre-incubated for 1 h with 1.0% polyethylenimine, and washed four times with ice-cold wash buffer [50 mM Tris/HCl (pH 7.4), 100 mM NaCl, 0.05% Tween-80]. Filters were then dried for 30 min before attachment to a MeltiLex A melt-on scintillation sheet (PerkinElmer Life and Analytical Sciences, Boston, MA, United States). The radioactivity remaining on the filter was monitored with a MicroBeta Trilux microplate scintillation counter (PerkinElmer Life and Analytical Sciences, Boston, MA, United States). Affinities at equilibrium were determined as antagonist dissociation constant (*K*i) values after correcting the experimentally determined IC_50_ values with the experimentally determined *K*d value of NMS for hM_2_R or hM_3_R and the concentration of NMS, as described previously (Yamashita et al., 2014). Shown values are an average of at least two independent experiments. The *K*i value was obtained from three independent curves. All curve-fitting procedures were performed using Prism (GraphPad Software, Inc., San Diego, CA, United States).

### Statistical Analysis

All values are expressed as the mean ± SEM. Homoscedasticity of data were verified by Levene test, then, data were examined using one-way ANOVA followed by the Tukey test or Games-Howell test for unpaired results was used to evaluate differences between three or more groups. Differences were considered to be statistically significant for values of *P* < 0.05.

## Results

### Affinity for hM_3_R and Anti-inflammatory Activities of **MP, TP, GC, and AD**

**FIGURE 1:**
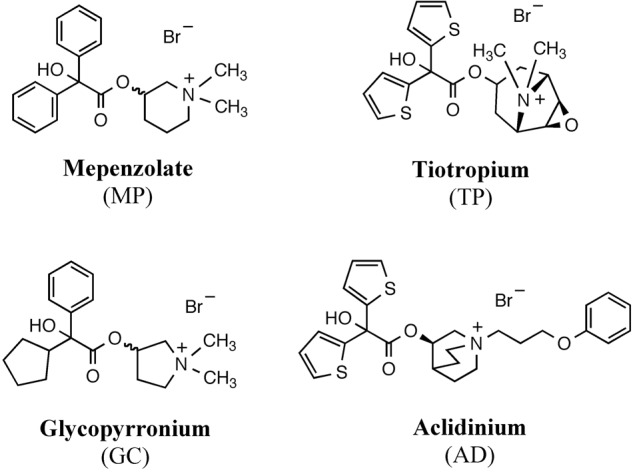
Structure of clinically-used muscarinic antagonists.

We first compared the binding of **MP, TP, GC, and AD** to hM_3_R and hM_2_R (**Figure 1**). The binding affinity of each compound to each receptor was determined by carrying out [^3^H]NMS displacement studies on each receptor. A higher *K*i value means a lower affinity for hM_3_R, because *K*i value is related to the concentration of each compound that causes 50% inhibition of NMS-binding to hM_3_R. As shown in **Table 1, MP** showed the highest *K*i value (by a factor of at least five compared with **TP, GC, and AD**), thus indicating it had the lowest affinity for hM_3_R. Similar results were observed for hM_2_R (**Table 1**).

**Table 1:** Binding of compounds to hM2R or hM3R.

Compound	*Ki* (nM)	
	hM_3_R	hM_2_R
Mepenzolate (MP)	4.6	1.8
Tiotropium (TP)	0.22	0.64
Glycopyrronium (GC)	0.7	1.0
Aclidinium (AD)	0.34	0.39
MP-GC	1.1	0.79
GC-MP	1.2	0.53
MP-AD	0.72	0.64

*Membrane fractions prepared from CHO-K1 cells expressing hM2R or hM3R were incubated with 2 nM [^*3*^H]NMS (85.5 Ci/mmol) in the presence of each compound. A range of concentrations (10^-*11*^ to 10^-*6*^ M) for each compound was tested in triplicate to generate competitive binding curves to determine *Ki* values. The shown value is an average of at least two independent experiments.*

The total number of leucocytes and the individual number of neutrophils in BALF are indicators of pulmonary inflammatory responses; these increased after PPE treatment in a manner that could be partially suppressed by the simultaneous intratracheal administration of **MP** (**Table 2**), as described previously (Tanaka et al., 2013, 2014). Because compared to neutrophils, neither the macrophage nor eosinophil number was so apparently increased in BALF after PPE treatment (Tanaka et al., 2013, 2014), we did not count them here. As none of **TP, GC, and AD** showed anti-inflammatory activity equivalent to that of **MP (Table 2**), we attempted to identify **MP** derivatives that had an affinity for hM_3_R equivalent to that of **TP, GC, and AD** and an anti-inflammatory activity equivalent to that of **MP**.

**Table 2:** Effect of intratracheal administration of compounds on PPE-induced inflammatory responses.

	Total cells (×10^5^ cells)	Neutrophils (×10^5^ cells)
Control	1.26 ± 0.25	0.12 ± 0.07
PPE	9.66 ± 0.54^∗∗^	7.46 ± 0.57^∗∗^
PPE + MP (37.5 μg/kg)	6.30 ± 0.90^##^	4.11 ± 0.68^##^
PPE + TP (8.4 μg/kg)	10.00 ± 1.27	6.81 ± 1.03
PPE + TP (42.1 μg/kg)	10.05 ± 0.57	7.31 ± 0.22
PPE + GC (7.1 μg/kg)	10.20 ± 0.93	7.34 ± 0.92
PPE + GC (35.5 μg/kg)	9.09 ± 1.09	6.25 ± 0.83
PPE + AD (10.1 μg/kg)	11.88 ± 2.01	9.06 ± 1.63
PPE + AD (50.4 μg/kg)	9.75 ± 1.12	6.86 ± 0.88

*Mice were treated with or without (control) PPE (20 U/kg) once only on day 0. The indicated doses (μg/kg) of each compound were administered intratracheally once only on day 0. Twenty-four hours after the PPE administration, BALF was prepared and the total cell number and the number of neutrophils were determined as described in Section “Materials and Methods.” Values represent mean ± SEM (*n* = 4–8). ^∗∗^*P* < 0.01 (versus control); ^##^*P* < 0.01 (versus PPE only). Data are representative of two independent experiments.*

### Synthesis of Hybrid Compounds and Their Affinities for hM_3_R

**FIGURE 2:**
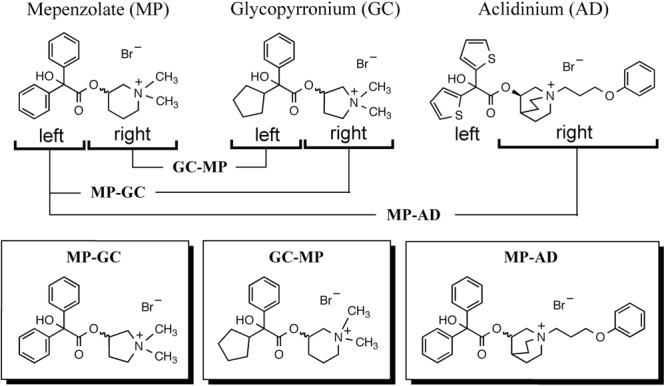
Structure of hybrid compounds. The chemical structure of each compound [mepenzolate (**MP**), glycopyrronium (**GC**), and aclidinium (**AD**)] was separated into parts left and right of the ester bond. These parts were then used to synthesize the shown hybrid structures.

**SCHEME 1:**
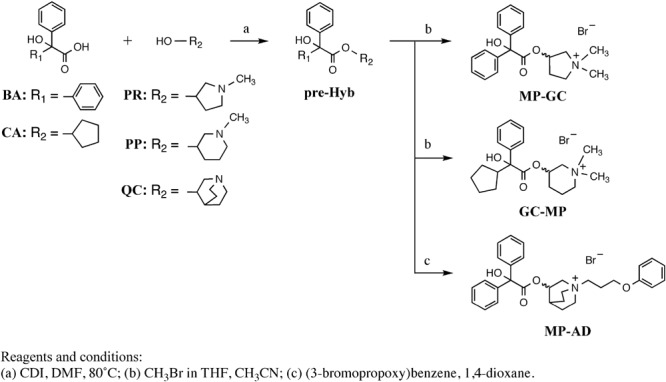
Synthesis of compounds **MP-GC, GC-MP, and MP-AD.**

We synthesized hybrid compounds based on **MP** and LAMAs used in the clinical setting (**GC** and **AD**). The structures of **MP** and these LAMAs were separated into two parts with a view to synthesizing hybrid compounds by substitution of each of the parts (**Figure 2**). We synthesized mepenzolate-glycopyrronium **(MP-GC)**, glycopyrronium-mepenzolate **(GC-MP)** and mepenzolate-aclidinium **(MP-AD)** (**Figure 2**). The synthetic route for these compounds are outlined in **Scheme 1**. First, the ester compound (**pre-Hyb**) was synthesized by the condensation of an appropriate carboxylic acid (**BA** or **CA**) and aminoalcohol (**PR, PP**, or **QC**). The precursor of target compound (**pre-Hyb**) was quaternized with the appropriate methyl bromide or phenoxy alkyl bromide to give target compounds (**MP-GC, GC-MP, and MP-AD**). These compounds are known and were identified by their melting points, NMR spectra, and mass spectra. On the other hand, we could not synthesize a hybrid compound based on aclidinium-mepenzolate.

As shown in **Table 1**, all of the hybrid compounds had a lower *K*i value for hM_3_R than **MP**. Of the target compounds, **MP-AD** showed the lowest *K*i value for hM_3_R (highest affinity for hM_3_R), while **GC-MP** showed the lowest *K*i value for hM_2_R.

### Bronchodilation Activities of Hybrid Compounds

**FIGURE 3:**
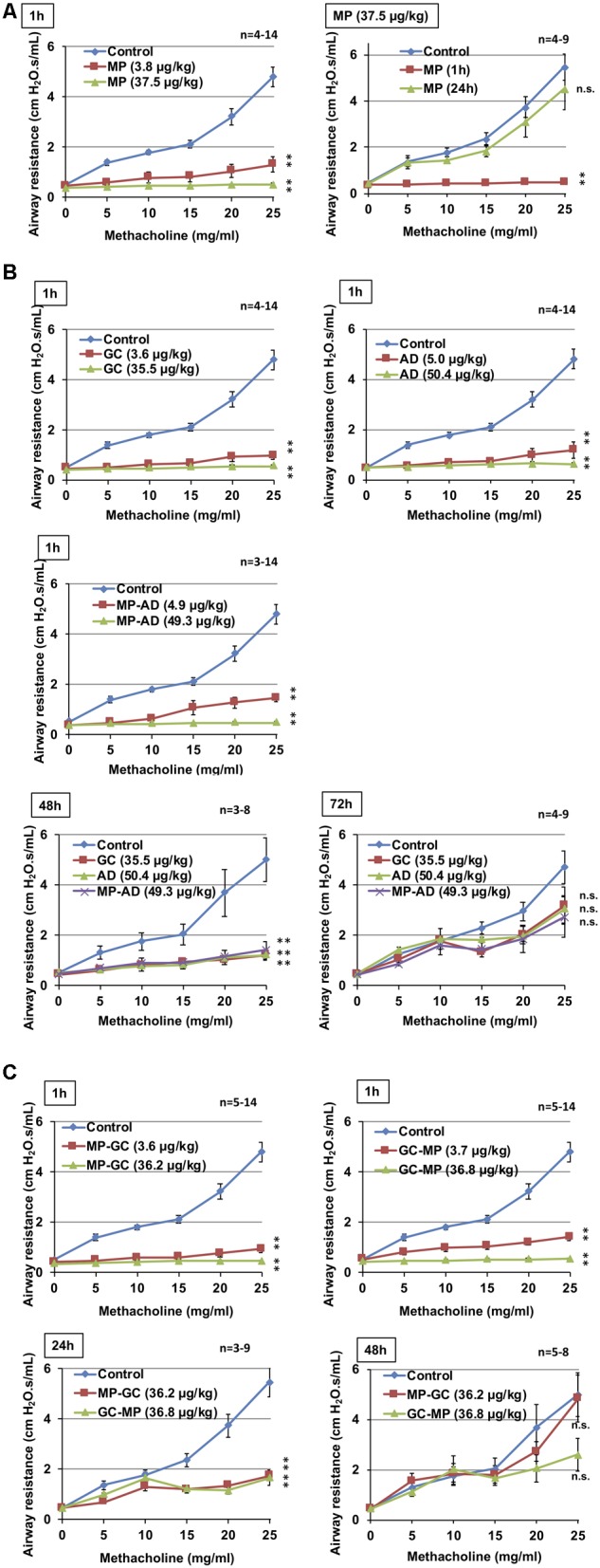
Effect of compounds on methacholine-induced airway constriction. Indicated doses of **MP (A), GC, AD**, and **MP-AD (B)** or **MP-GC** or **GC-MP (C)** were administered intratracheally. After the indicated period, mice were exposed to nebulized methacholine 5 times and airway resistance was determined after each methacholine challenge as described in Section “Materials and Methods.” Values represent mean ± SEM. ^∗^*P* < 0.05; ^∗∗^*P* < 0.01; n.s., not significant. The control data is the same in all graphs. Data are representative of two independent experiments.

As shown in **Figure 3A**, the intratracheal administration of **MP** induced bronchodilation in a dose-dependent manner, which is consistent with our previously reported findings (Tanaka et al., 2013, 2014). We also confirmed that the bronchodilatory activity of **MP** disappeared within 24 h (**Figure 3A**), suggesting that its effects were short-acting.

We then compared these results with the duration of bronchodilatory activity of **GC, AD**, and **MP-AD**. As shown in **Figure 3B**, these compounds all exhibited dose-dependent bronchodilatory activity within 1 h of their administration. Compared to baseline, significantly increased bronchodilation (reduced airway resistance) was observed 48 h (but not 72 h) after the administration, suggesting that **MP-AD** has a long-lasting bronchodilatory activity, similar to that of **GC** and **AD**. We also examined the duration of bronchodilation for **MP-GC** and **GC-MP**. Both of these compounds showed bronchodilatory activity at 1 and 24 h, but not at 48 h (**Figure 3C**), suggesting that **MP-GC** and **GC-MP** exhibit bronchodilatory activity intermediate to that of **MP** and **MP-AD**.

### Anti-inflammatory and Anti-emphysemic Activities of Hybrid Compounds

**FIGURE 4:**
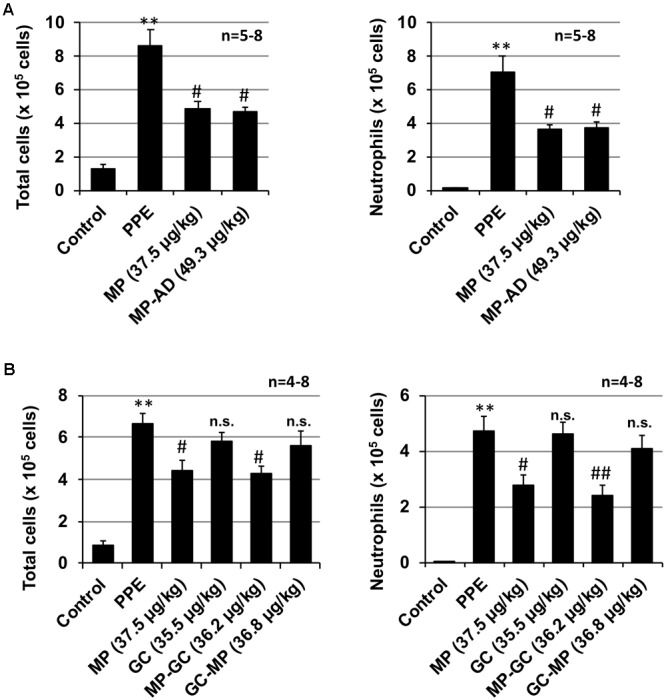
Effect of compounds on PPE-induced inflammatory responses. Mice were treated with or without (control) PPE (20 U/kg) once only on day 0. The indicated doses (μg/kg) of **MP** and **MP-AD (A)** or **MP, GC, MP-GC**, or **GC-MP (B)** were administered intratracheally once only. Six hours after the PPE administration, BALF was prepared and the total cell number and the number of neutrophils were determined as described in Section “Materials and Methods.” Values represent mean ± SEM. ^∗∗^*P* < 0.01 (versus control); ^#^*P* < 0.05, ^##^*P* < 0.01 (versus PPE only); n.s., not significant. Data are representative of two independent experiments.

As shown in **Figure 4A**, the anti-inflammatory activity of **MP-AD** was similar to that of **MP**. We also found that **MP-GC** exhibited anti-inflammatory activity, but as for **GC, GC-MP** did not (**Figure 4B**). These results suggest that the double phenyl rings in **MP** are important for its anti-inflammatory activity.

**FIGURE 5:**
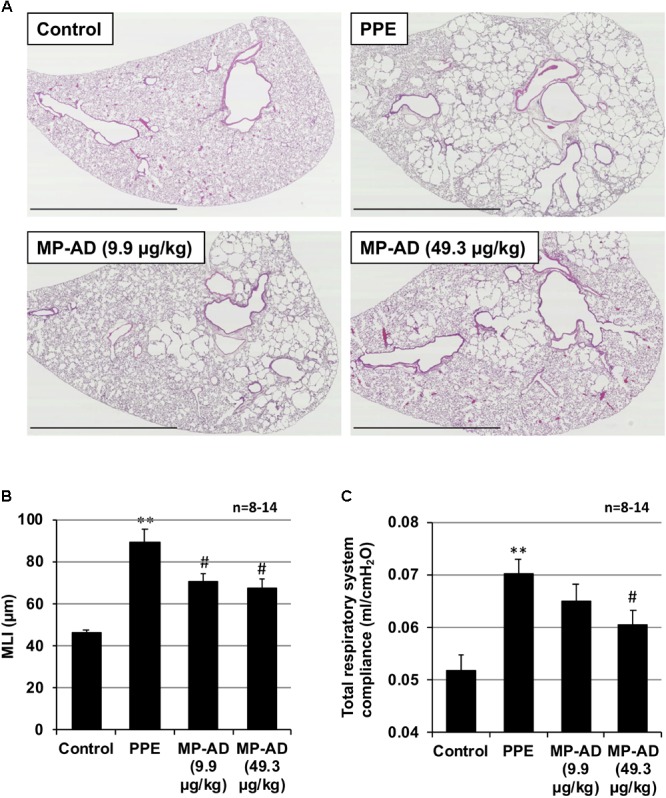
Effect of **MP-AD** on PPE-induced pulmonary damage. Mice were treated with or without (control) PPE (15 U/kg) once only on day 0. The indicated doses of **MP-AD** were administered intratracheally once daily for 16 days (from day 0 to day 15). Sections of pulmonary tissue were prepared on day 21 and subjected to histopathological examination (H&E staining) (scale bar: 2.5 mm). **(A)** Airspace size was estimated by determining the MLI as described in Section “Materials and Methods.” **(B)** Total respiratory system compliance was determined on day 21 as described in Section “Materials and Methods.” **(C)** Values represent mean ± SEM. ^∗∗^*P* < 0.01 (versus control); ^#^*P* < 0.05 (versus PPE only). Data are representative of two independent experiments.

We next examined the effect of the long-term (up to 15 days) administration of **MP-AD** on PPE-induced lung injury. Histopathological analysis revealed that while PPE administration damaged the alveolar walls and increased MLI, this effect could be partially suppressed by the administration of **MP-AD** (**Figures 5A,B**). We could not find any adverse effects (such as decrease in body weight or behavior abnormalities) in mice subjected to the long-term administration of **MP-AD**. Treatment of mice with PPE increased total respiratory system compliance (**Figure 5C**), as described previously (Hamakawa et al., 2011). We here found that treatment of mice with **MP-AD** restored total respiratory system compliance (**Figure 5C**). Based on these results, we suggest that **MP-AD** suppresses PPE-induced emphysema through its anti-inflammatory activity.

## Discussion

To obtain **MP** derivatives with longer-acting bronchodilatory activity, we synthesized hybrid compounds based on **MP** and **GC** or **AD**. Among the compounds identified (**MP-GC, GC-MP, and MP-AD**), **MP-AD** showed the highest affinity for hM_3_R and the longest bronchodilatory activity. It should be noted that the duration of bronchodilation associated with **MP-AD** is equivalent to that of **GC** and **AD**, which are clinically used LAMAs. Based on these results, we speculate that the *N*-substituted 3-quinuclidinyl ester of **AD** is important for its long-acting bronchodilatory properties. On the other hand, both **MP-GC** and **MP-AD** showed anti-inflammatory effects similar to **MP**, whereas **GC-MP**, like **GC** and **AD**, did not exhibit this effect. Based on these results, we speculate that the double phenyl rings in **MP** are important for its anti-inflammatory activity. Our findings thus provide important information concerning the structure-activity relationship for **MP**, which underlies its bronchodilatory and anti-inflammatory activities. We consider that *in silico* drug design should be done for this target, because such trial for other diseases was successfully done for other studies (Corbo et al., 2007; De Luca et al., 2012). Such structure-activity investigation may also help to identify compounds with better toxicological profile avoiding off target effects (De Bellis et al., 2017).

As for the mechanism underlying these effects, the results from this study support the notion that the bronchodilatory effect is mediated through an antagonistic action toward hM_3_R. Concerning the anti-inflammatory effects seen here, we previously showed that **MP** decreases ROS levels in the lung by inhibiting NADPH oxidase activity and by inducing the expression of SOD and GST, both of which are endogenous anti-oxidants (Tanaka et al., 2013). Furthermore, we previously described inhibitory effects of **MP** on nuclear factor-κB and histone deacetylase 2, both of which are involved in the pathogenesis of COPD (Tanaka et al., 2013). Although we did not examine the effect of **MP-AD** on these inflammation-related proteins, a similar mechanism may underlie the anti-inflammatory properties of **MP-AD**. In order to confirm this, immunohistochemical experiments would be useful.

We measured airway resistance in mice exposed to nebulized methacholine, which is the most representative method for monitoring bronchodilatory effects (Ather et al., 2016; Murad et al., 2017). Although this method is convenient, it seems to be an asthmatic-like condition rather than COPD-like condition. Therefore, the effect of each drug on airway resistance should be examined in COPD animal model in future. Furthermore, in emphysema, the most important parameter is a compliance of pulmonary alveolus or total respiratory system. The compliance correlates best with lung structure (structural parameters) (Hamakawa et al., 2011). We here showed that treatment of mice with PPE increased total respiratory system compliance and that treatment of mice with **MP-AD restored** total respiratory system compliance (**Figure 5C**).

In this study, we used intratracheal administration of each drug, because this method is convenient for experiments. However, it is possible that the compound does not distribute evenly through this method (Tanaka et al., 2010; Sato et al., 2015). The patchy appearance of airspace enlargement on the histological section following treatment with **MP-AD** may be explained by this uneven distribution of the drug. Thus, we should examine the distribution of this drug in the lung after intratracheal administration in future.

We also confirmed here that **MP-AD** suppressed elastase-induced pulmonary damage and emphysema in a COPD mouse model. Taken together, we propose that **MP-AD** could serve as a new drug candidate for the treatment of COPD, given its long-lasting bronchodilatory and anti-inflammatory activity.

## Author Contributions

K-IT, NY, YY, YF, and TM: participated in research design. K-IT, NY, YY, TA, YK, and AT: conducted experiments. MK, MT, and YF: contributed new reagents or analytic tools. K-IT, NY, YY, TA, and TM: performed data analysis. K-IT, NY, YY, TA, YK, AT, MK, MT, YF, and TM: wrote or contributed to the writing of the manuscript.

## Conflict of Interest Statement

LTT Bio-Pharma Co., Ltd. is developing **MP-AD**-related compounds (such as **MP**) as drugs for the treatment of COPD. TM is Chairman of LTT Bio-Pharma Co., Ltd. TA and MT are employees of an endowed research division of LTT Bio-Pharma Co., Ltd. The other authors declare that the research was conducted in the absence of any commercial or financial relationships that could be construed as a potential conflict of interest.
